# Automated diagnostic method for sleep apnea and hypopnea using overnight airflow and oxygen saturation

**DOI:** 10.1016/j.mex.2025.103528

**Published:** 2025-07-27

**Authors:** Yi Yu, Jing-Jing Huang, Hui Yang, Liu-Jie Ren

**Affiliations:** a College of Medical Instruments, Shanghai University of Medicine & Health Sciences, Shanghai, China; b ENT institute, Eye & ENT Hospital, Fudan University, Shanghai, China; c Sleep Medicine Center, Eye & ENT Hospital, Fudan University, Shanghai, China; d FPRS Department, Eye & ENT Hospital of Fudan University, Shanghai, China

**Keywords:** Local range analysis, Blood oxygen desaturation, Apnea event detection, Home-based monitoring, Signal thresholding, Sleep disorder screening, Machine-assisted scoring

## Abstract

Polysomnography (PSG) is the gold standard for diagnosing obstructive sleep apnea-hypopnea syndrome (OSAHS), but it relies heavily on manual scoring by experienced clinicians, which is time-consuming and costly. This study presents an automated method for detecting sleep apnea and hypopnea events using overnight airflow and blood oxygen saturation signals. Apnea episodes are identified through local range (LR) analysis of airflow, while rapid oxygen desaturation supports hypopnea detection. LR thresholds were trained on data from 100 patients and validated on an independent cohort of 43 patients. The system’s performance was compared to clinician annotations using the apnea-hypopnea index (AHI). The results demonstrated high consistency with manual scoring, highlighting the method’s potential for home-based monitoring and large-scale screening. This approach offers a lightweight and scalable alternative to traditional diagnostic procedures, reducing dependence on complex equipment and human interpretation.

## Highlights

• A lightweight algorithm using local range analysis to detect apnea events from airflow signals.
• Oxygen desaturation-based enhancement improves hypopnea detection accuracy.
• Validated on 43 patients with strong agreement to clinician-annotated AHI values.

## Graphical abstract

**Figure:**
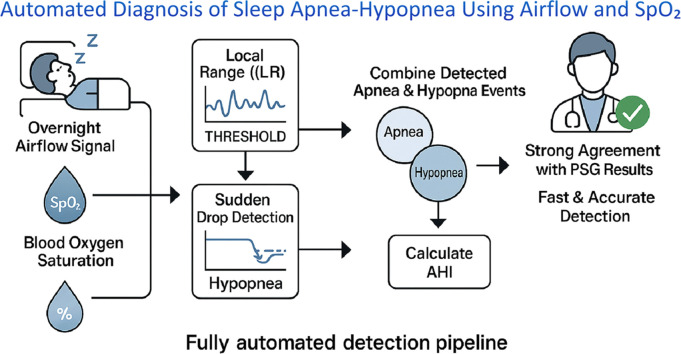

**Specifications table**

**Table:** 

**Subject area**	Engineering
**More specific subject area**	Sleep disorder diagnosis; Biomedical signal processing
**Name of your method**	Automated Detection of Sleep Apnea and Hypopnea Events Using Airflow and Blood Oxygen Signals
**Name and reference of original method**	Not applicable
**Resource availability**	Patient data are provided in the appendix. Algorithm implementation code is available upon request from the corresponding author.

## Background

Sleep apnea-hypopnea syndrome (SAHS) is an increasingly prevalent sleep-related breathing disorder that poses a major risk to public health. It is characterized by repeated episodes of partial (hypopnea) or complete (apnea) obstruction of the upper airway during sleep. Apnea events are defined as complete cessation of airflow for at least 10 s, while hypopnea involves a significant reduction in airflow, often >50 %. These episodes disrupt sleep continuity and result in poor sleep quality, leading to daytime symptoms such as fatigue, excessive sleepiness, impaired concentration, and increased risk of accidents [1]. Long-term untreated SAHS has been associated with serious comorbidities, including hypertension, cardiovascular disease [2], stroke, and even sudden cardiac death. Despite the high prevalence and health impact of SAHS, studies estimate that up to 80 % of moderate-to-severe cases remain undiagnosed [3].

The current gold standard for SAHS diagnosis is polysomnography (PSG), a comprehensive overnight test that records multiple physiological signals [4], including airflow, electrocardiogram (ECG), blood oxygen saturation (SpO_2_), and snoring [5]. Clinicians interpret these signals manually to identify apnea and hypopnea events. This process is labor-intensive, requires significant clinical expertise, and typically takes 90 to 120 min per patient. The diagnostic outcome is summarized using the apnea-hypopnea index (AHI) [6], which represents the average number of apnea-hypopnea events per hour of sleep. According to clinical guidelines, an AHI between 5 and 15 is classified as mild SAHS, 15 to 30 as moderate, and 30 or more as severe.

In recent years, growing interest in sleep medicine and advances in computational analysis have led to the development of alternative diagnostic methods that rely on a reduced set of PSG signals [[7], [8], [9]]. Prior research has explored the potential of individual or combined signals—such as oxygen saturation signals [10], airflow [[11], [12], [13], [14], [15]], ECG [16], EEG [17], SpO_2_ data [[18], [19], [20]], or combinations of these signals [21,22] for automating the detection of SAH events. Many of these approaches aim to reduce diagnostic time and enable home-based or large-scale screening.

According to the American Academy of Sleep Medicine (AASM) [23], the presence of a SAH event can be determined by *a* > 50 % reduction in breathing or a smaller reduction accompanied by either oxygen desaturation (>3 %) or arousal. Therefore, airflow and SpO_2_ are considered the most relevant signals for SAHS event detection. In this context, the development of automated methods using these two signals can provide a simpler, more scalable alternative to PSG, especially in resource-limited or home-care settings.

The present study introduces an automated method that detects apnea events by analyzing the local range of airflow signals and supports hypopnea detection through rapid desaturation patterns in SpO_2_ signals. The method is developed using clinical data and evaluated by comparing its output with manually annotated clinical results in terms of SAH event count, AHI, and event duration. This methodology is intended to address the need for efficient, accessible, and reliable SAHS screening solutions.

## Method details

A total of 143 patients’ data were retrospectively collected (122 males and 21 females, mean age: 40.8 years, from July 1st, 2019 to October 30th, 2019) from the Eye & ENT Hospital of Fudan University. These patients complained of snoring or daytime sleepiness. To protect patient privacy, all data were anonymized throughout the entire analysis process after collection.

The overnight airflow and SpO_2_ records were employed, which were obtained and exported from the clinically used PSG system (Embla N7000, Natus, USA). The flow data were stored as a time series with 0.005-s intervals (i.e., a sampling rate of 200 Hz), and the SpO_2_ data were stored as a time series with 0.1-s intervals (i.e., a sampling rate of 10 Hz). Additional data, such as sleep monitoring data (sampling rate: 1/30 Hz), manually marked SAH events (to avoid personal bias, the data were manually marked by 5 different clinicians), the event durations, and clinical reports of *AHI*, were also collected for analysis.

The patients were randomly divided into two groups: the training group (100 people) and the test group (40 people). The training group was used to determine the parameters for the automated procedure to detect events, as will be discussed later. After parameter optimization, the procedure was tested using the test group.

### Detecting apnea events using the airflow

The airflow waveform is directly affected by the occurrence of respiratory events [24]. Clear oscillations are observed during normal breathing periods, whereas apnea and hypopnea cause a noticeable amplitude reduction. Therefore, an in-depth analysis of the information from the single-channel airflow was proposed to assist in SAHS diagnosis. There are many methods based on airflow, such as spectral analysis [11], envelope detectors [1], nonlinear methods [11], Hilbert-Huang decomposition [12] and machine learning-based methods (such as multiple-layer perceptron [14] and boosting algorithm [15]).

In this study, the local range (*LR*) was adopted to evaluate the airflow pattern. *LR* is defined as the difference in value between the local maximum and minimum of an airflow segment wave P(t),

LR(t)=max(P(t→t+Δt))−min(P(t→t+Δt))

Here, LR(t) is the local range at time t, and P(t→t+Δt) is the airflow segment from time t to t+Δt. The segment length Δt was chosen to be 10 s (the clinical minimum duration of detecting breathing events).

**Fig. 1:**
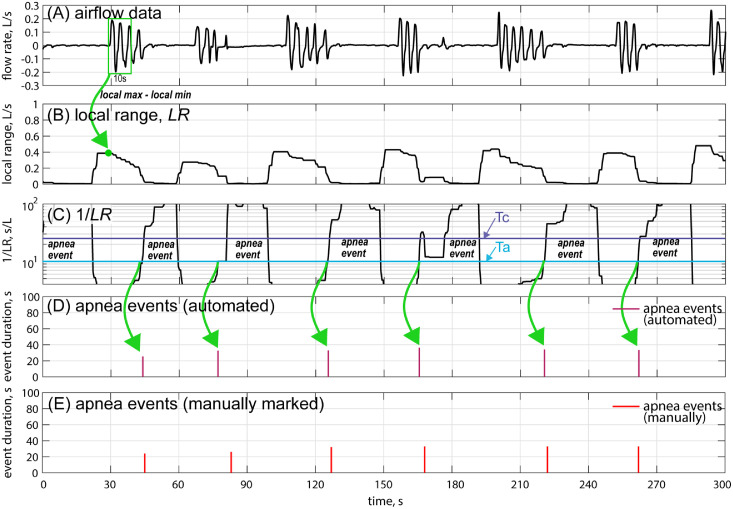
Apnea event detection based on the local range of airflow. (A) A segment of the airflow data lasting 5 min. (B) Local range (*LR*) calculated every 0.5 s. (C) 1/*LR* is used as the criterion to detect apnea events according to thresholds $T_{a}$ and $T_{c}$. (D) Automated diagnosed apnea events according to *LR*. (E) Apnea events manually marked in the clinical reports.

Fig. 1 gives an example of the detection of apnea events using LR. Fig. 1(A) shows a segment of the original flow data, and Fig. 1(B) shows the corresponding LR (calculated every 0.5 s). It demonstrates that LR becomes relatively small when an apnea event occurs. To better illustrate the strong correlation, 1/LR is plotted in Fig. 1(C) alongside clinically marked apnea events (Fig. 1(E)): 1/LR peaks whenever an apnea event occurs.

### *LR* thresholds

To determine an apnea event using LR (or more precisely, 1/LR), two thresholds, $T_{a}$ and $T_{c}$ ($0<T_{a}<T_{c}$), are introduced (see Fig. 1(C) and (D)). $T_{a}$ is the threshold to distinguish between a single apnea event and multiple apnea events. If 1/LR>$T_{a}$ at several dense peaks, they are considered a single apnea event. $T_{c}$ is the threshold used to determine if an event qualifies as an apnea event. Thus, if $1/LR>T_{c}$, it is considered an apnea event.

**Fig. 2:**
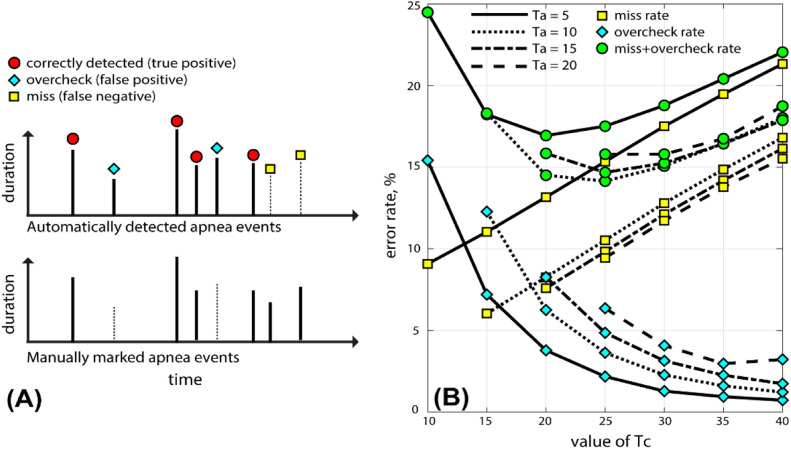
Error rate of apnea event detection for different *T_a_* and *T_c_*. (A) Schematic diagram of “overcheck” and “miss” detection. (B) The dependence of the overcheck rate, miss rate, and their sum on *T_a_* and *T_c_*.

A total of 100 airflow data points (from the training group) were used to determine the optimal combination of $T_{a}$ and $T_{c}$, and error estimation was conducted, with the results shown in Fig. 2. As shown in Fig. 2(A), the “overcheck” rate defines the rate at which normal breathing is incorrectly detected as an apnea event (a false positive), while the “miss” rate defines the rate at which an apnea event is misclassified as normal breathing and is not detected (a false negative). The “overcheck” and “miss” rates at different value combinations are plotted in Fig. 2(B). When $T_{a}=10$ and $T_{c}=25$, the sum of the overcheck and miss rates is minimized.

Fig. 1(D) shows the automated detection of apnea events. Compared to the manually marked clinical reports (Fig. 1(E)), these events are correctly detected. Moreover, to evaluate the duration of the apnea events, the following approximation formula was used:

$$\Delta T_{\text{apnea}}=\Delta T_{\frac{1}{LR}>T_{a}}+10\;\text{seconds}$$

where $\Delta T_{\text{apnea}}$ is the duration of the apnea event, and $\Delta T_{\frac{1}{LR}>T_{a}}$ is the time duration when $\frac{1}{LR}>T_{a}$ during this event.

### SpO_2_ drops and breathing events

Theoretically, airflow data can also be used to diagnose hypopnea events, because a significant characteristic of hypopnea events is a relative drop in airflow amplitude, i.e., by 50 %. However, in practice, this detection is problematic. First, it is more challenging to find a convincing criterion or an effective algorithm that can reliably separate hypopnea events. Moreover, the sensitivity of the airflow baseline to the patient’s sleep posture and the position of the sensor adds complexity to event detection. Huang et al. [25] proposed a linear interpolation of current and historical baselines to estimate the time-dependent normal breathing airflow baseline; however, the coefficient values they used were not published. In clinical practice, it is often difficult and ambiguous for an expert to detect whether a hypopnea event has occurred using airflow data alone. Instead, clinicians usually rely heavily on SpO_2_ data; i.e., the patient’s blood oxygen saturation, which can be influenced by breathing disorders (SAH events), and is characterized by sudden drops.

**Fig. 3:**
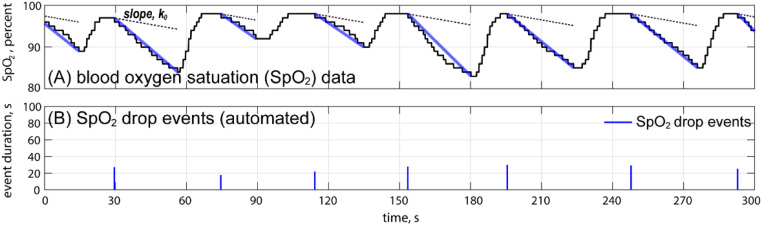
Detection of SpO_2_ drop events. (A) A section of an SpO_2_ data sample lasting 5 min. (B) Detected drop events.

Fig. 3(A) shows a typical segment of SpO_2_ data (denoted as Q(t)) lasting 5 min. The SpO_2_ data range from 0 % to 100 %. In this study, a “sudden drop” event was marked when two simple rules were satisfied: (1) the SpO_2_ data dropped by at least 3 %, i.e., $Q\left(t_{1}\right)-Q\left(t_{2}\right)\geq 3\%$, and (2) the drop slope was greater than a certain value, i.e., $\frac{Q\left(t_{1}\right)-Q\left(t_{2}\right)}{t_{2}-t_{1}}>k_{0}$. Here, $k_{0}=0.001$ was adopted.

The bold lines in Fig. 3(A) represent the detected “sudden drop” events, with the thin dotted lines showing a line of slope $k_{0}$. The durations of the drop events are determined as $t_{2}-t_{1}+t_{0}$, where $t_{0}$ is the lasting time of the SpO_2_ valley. The accordingly detected drop events are plotted in Fig. 3(B).

### Merging data and automated diagnosis

It should be clarified that SpO_2_ drop events can be induced by both apnea and hypopnea events. Since apnea events are already detected using airflow data, the remaining drops should be attributed to hypopnea events. Based on this simple principle, events detected from both airflow and SpO_2_ data were merged to obtain the final detection.

The main issue in merging the two sets of data is that SpO_2_ drops are typically delayed. Moreover, the delay time is patient-specific. However, it was observed that the SpO_2_ drop generally occurs within the following time period: from the timestamp when the event starts to the timestamp when the event ends, plus 30 s. Therefore, the following rules were adopted. For each SpO_2_ drop event, we looked back for up to 30 s. If at least one apnea event (detected from the airflow data) was found, the drop event was discarded. Otherwise, the drop event was classified as a hypopnea event.

**Fig. 4:**
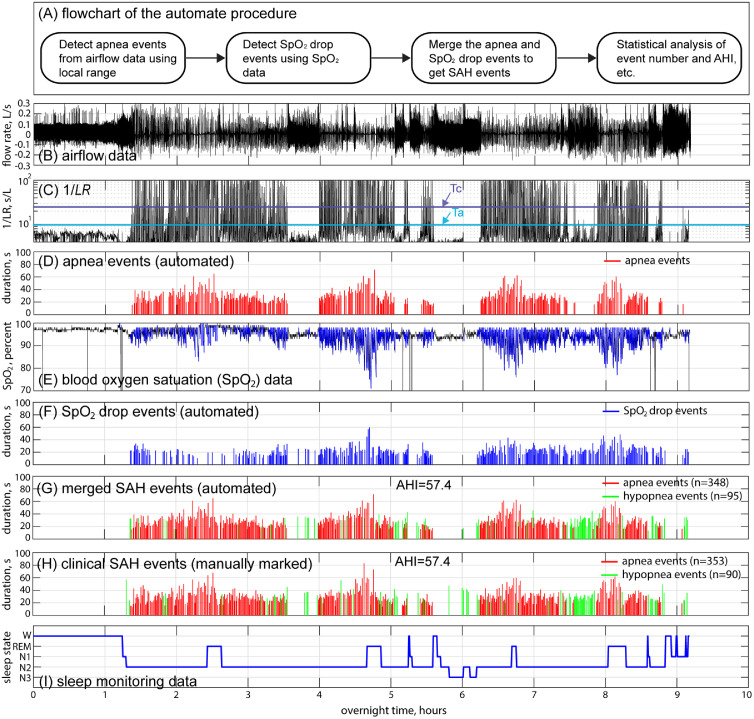
The automated diagnostic procedure. (A) The flowchart of the automated procedure. (B-I) An example showing the automated diagnostic process.

Overall, the automated diagnostic procedure became clear, as shown in Fig. 4(A). Fig. 4(B-I) provides an example of this procedure. First, apnea events were detected using the *LR* of airflow data (see Fig. 4B to D). Second, the sudden drop events of SpO_2_ were identified based on simple rules, as discussed earlier (see Fig. 4E and F). Then, the SpO_2_ drop events were merged with the previously detected apnea events, excluding overlapping events, and the remaining events were classified as hypopnea events (see Fig. 4G for the merged SAH events; the manually marked SAH events are also shown in Fig. 4H for comparison). Finally, statistical data, such as the event numbers and *AHI* data of the patients, were automatically calculated to generate a brief report.

### Event number and *AHI* calculation

Since the diagnosis of SAHS in clinical practice is based on the *AHI*, which is defined as:

$$AHI=\frac{n_{SAH}}{T}=\frac{n_{A}+n_{H}}{T}$$

where $n_{SAH}$ is the number of SAH events; $n_{A}$ and $n_{H}$ are the numbers of apnea and hypopnea events, respectively; and T is the total sleep time. The number of SAH events can be obtained through the automated diagnostic procedure, as described previously. The total sleep time can also be obtained. One way to calculate the total sleep time is by using the EEG data (as shown in Fig. 4(I)). The AASM stipulates that the sleep period of adults is divided into periods of wakefulness (*W*), non-rapid eye movement (including NREM 1 (*N1*), NREM 2 (*N2*), NREM 3 (*N3*)) and REM (*R*) [23]. The total sleep time can thus be calculated as:

Totalsleeptime=N1+N2+N3+R

Therefore, the total sleep time can be automatically calculated.

### Error analysis and statistics

To better evaluate the automated procedure, event-by-event error analysis was conducted in comparison with manually marked results. Since apnea and hypopnea events contribute equally to *AHI*, the most important diagnostic criterion, we did not deliberately distinguish them. Two types of errors were considered, as described earlier: (1) an “overcheck” (false positive), when a non-event is detected as an SAH event and (2) a “miss” (false negative), when an SAH event is not detected.

In addition, statistical results were compared, such as the *AHI* (SAH events per hour), apnea or hypopnea events per hour, etc. Ideally, the automated diagnostic results should match the clinically marked results. Therefore, the commonly used root mean squared error (*RMSE*) in regression analysis was adopted:

$$RMSE=\sqrt{\frac{\sum_{i}^{N}{\left(X_{i}^{\text{automate}}-X_{i}^{\text{manual}}\right)}^{2}}{N}}$$

where N is the number of samples (patients); *X* is the compared variable; and $X_{i}^{\text{automate}}$ and $X_{i}^{\text{manual}}$ are the results calculated from the automated procedure and clinical reports for patient i, respectively.

## Method validation

All analyses were performed on a personal laptop (Intel i7–8650 U, 16 GB RAM), with the protocol code was written and executed in MATLAB (R2020a). The average time required for analyzing the overnight airflow and SpO2 data per patient was under 3 s, including the processing of raw ASCII data files of approximately 90–100 MB.

### SAH event detection

Fig. 4 (B-I) gives the complete diagnostic procedure for the analysis of the overnight flow data (approximately 9 h) from a typical patient. By comparing Fig. 4(G) and (H), the automated diagnosis and clinical reports generally demonstrated good agreement in terms of event locations, distributions and durations. Statistically, the number of detected events was also similar: 348 apnea events and 95 hypopnea events were diagnosed, while the manually marked numbers were 353 and 90, respectively. The *AHI* values were also very close (57.4).

**Fig. 5:**
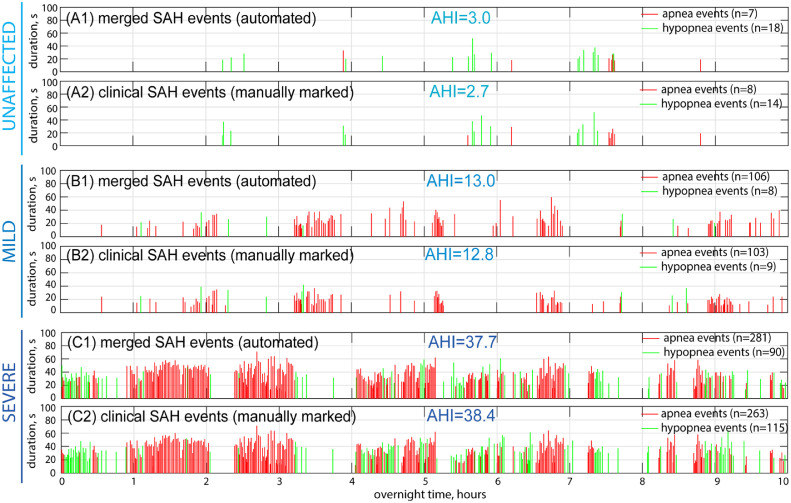
Examples of the SAH events in the automated diagnostic procedure for the diagnosis of SAHS: (A1-A2) unaffected person; (B1-B2) mild SAHS; (C1-C2) severe SAHS.

Three additional examples are shown in Fig. 5: one unaffected person, one with mild SAHS and one with severe SAHS. The results indicate that the automated diagnosis generally agreed with the manually marked results. Although errors occasionally occurred in terms of event-by-event correlation, statistically, the event numbers and *AHI* remained comparable (e.g., Fig. 5(B1-B2)).

Table 1 gives the event-by-event checked results for SAH events. The total number of SAH events for the analyzed 145 patients was 54,148 according to clinical reports, and 52,753 calculated from the automated procedure. The miss rate and error rate were approximately 7.0 % and 4.4 %, respectively. The sensitivity was 93.0 %, and the positive predictive value (PPV) was 95.5 %. No significant difference was observed between the training group and the test group. These “miss” and “overcheck” errors may have been caused by multiple factors: (1) The SAH events may have been ambiguous, making them difficult for both machines and clinicians to accurately judge; (2) Occasionally, there was divergence in the judgement of two separated events or one combined event; (3) Errors also occurred in linking an SpO_2_ drop event and the corresponding breathing event; (4) At times, the event was reflected by other channels; (5) Rarely, but potentially, clinicians made errors due to misjudgments or mismanipulations.

**Table 1:** Event-by-event error estimation for SAH events.

Groups	Total SAH events (manually marked)	Detected events True positive (number/rate)	Miss events False negative (number/rate)	Overcheck events False positive (number/rate)	Total SAH events (automate)
Training group (*N* = 100)	37,575	34,965/93.05 %	2610/6.95 %	1614/4.30 %	36,579
Test group (*N* = 43)	16,573	15,398/92.91 %	1175/7.09 %	776/4.68 %	16,174
Total (*N* = 143)	54,148	50,363/93.01 %	3785/6.99 %	2390/4.41 %	52,753

Combining airflow and SpO_2_ for SAHS diagnosis generally yields accurate results [8], although different datasets were used for testing, and the results could not be directly compared, as illustrated by the works of Tian and Liu (2005), Otero et al. (2012) and Huang et al. (2017) [[25], [26], [27]]. Tian and Liu also attempted to distinguish between apnea and hypopnea events; achieving a sensitivity was 83.7 % [26]. Otero et al. applied fuzzy rules and attained a good accuracy of 90 % [27]. Huang et al. achieved a high sensitivity score of 97.6 %, although this was based on only 30 patients [25].

Some of the basic detection approaches used in this study are similar to those employed by Huang et al. [25], though this similarity is purely coincidental. Both studies utilized the *LR* for fast detection of SAH events (referred to as Vpt in Huang’s paper), although it was proposed earlier [[28], [29]]; however, the subsequent detection rules differ significantly. Both studies used SpO_2_ data for detecting hypopnea events. The main difference is that Huang et al. tried to exclude non-events based on a previous check of hypopnea events from airflow, while in this study, we incorporated some unchecked SpO_2_ drop events as hypopnea events. According to statistical analysis, both the “subtraction rule” and the “addition rule” perform accurately and reliably.

### Statistical results – *AHI*, event numbers and duration

**Fig. 6:**
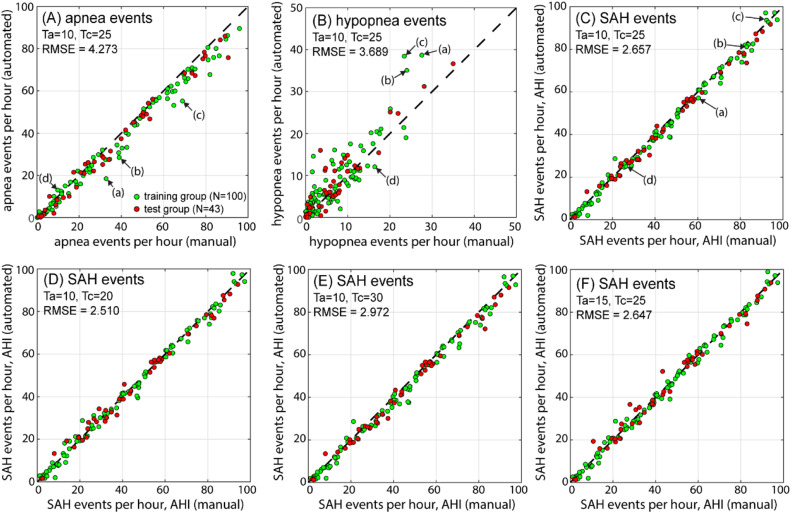
Comparison of event numbers (per hour) between the automated procedure and clinical manually marked results. (A) Apnea events per hour; (B) hypopnea events per hour; (C) SAH events per hour, *AHI*; (D to F) comparison of *AHI* values at different threshold combinations.

As single SAH event detection heavily relies on the responsible clinician’s experience, the detection is strongly subjective. However, statistically analysed results could be effective and reliable in the diagnosis of SAHS. Here, the detected event numbers per hour for each patient are plotted in Fig. 6, subfigure (A), for apnea events, (B) for hypopnea events and (C) for SAH events together (namely, the *AHI* value). Each dot, either from the training group or from the test group, denotes the diagnostic results for one patient: the x-axis gives the clinically reported result, and the y-axis gives the automated calculation. The closer the dots distribute along the dashed lines (y=x), the better the automated procedure performed. For detailed diagnostic data for each patient, readers may refer to the Appendix. For the number of apnea events (see Fig. 6(A)), the procedure performed quite well (*RMSE*=4.273). Unfortunately, the hypopnea events seem to not agree well (*RMSE*=3.689, see Fig. 6(B)). Nevertheless, the correlation between the automated diagnosed *AHI* and the clinical reports (see Fig. 6(C)) was surprisingly satisfying, with an even smaller *RMSE* (2.657) than that of the apnea events.

However, the above phenomena may be reasonable when a deeper look is taken. First, the number of hypopnea events is generally less than that of apnea events; as a result, some accidental errors for the hypopnea events will not influence much the total SAH events much. Second, and more importantly, it was shown that the number of apnea events diagnosed by the automated method was less than that diagnosed in the clinical reports, and the hypopnea event detection somehow compensated for this, which added to the accuracy of total SAH event detection. Approximately 2100 apnea events (accounting for 4.6 % of 45,735 apnea events in total) were diagnosed as hypopnea events. The labelled patients (a), (b) and (c) are three examples showing this compensation. The opposite case (more apnea events are detected with less detection of hypopnea events) seems to be rare but did exist, such as for patient (d). Compensation and “negative” compensation are essential, since, as will be shown later, it was rather inaccurate to estimate the *AHI* using the diagnosed apnea events only or, alternatively, to estimate the *AHI* using the SpO_2_ drop events only.

Optimized values of the thresholds $T_{a}$ and $T_{c}$ guarantee the performance of the automated procedure. It was found that the procedure worked well and robustly near the optimized values ($T_{a}=10,T_{c}=25$). Fig. 6 also gives diagnosed *AHI* results for different threshold values (D: $T_{a}=10,T_{c}=20$; E: $T_{a}=10,T_{c}=30$; F: $T_{a}=15,T_{c}=25$). The *AHI* calculations were also very close to those of the clinical reports. The combination $T_{a}=10,T_{c}=20$ showed even better performance (*RMSE*=2.510).

**Fig. 7:**
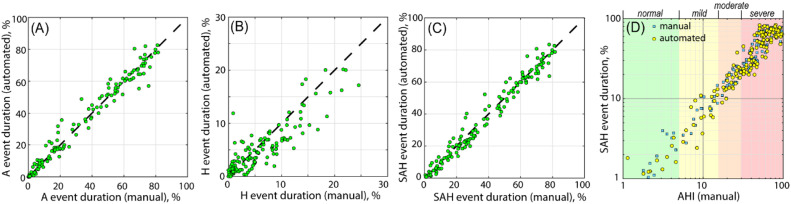
Event duration percentage and its relation with *AHI*. (A-C) Comparison between automated and clinical results of apnea (A), hypopnea (H) and both event duration percentages. (D) The relation between the SAHS event duration percentage and the *AHI*.

Fig. 7(A-C) plots the event duration (subfigure (A) for apnea events, (B) for hypopnea events and (C) for both events and the percentage for each patient. The SAH event duration was denoted as the sum of all single-event durations, and the percentage the event takes of the total sleep time was calculated as a potential new diagnostic parameter. This parameter was proposed based on the simple idea that the longer these SAHS events lasted, the more harmful effects they caused to the patient. In Fig. 7(D), the relation between the *AHI* and the SAH event duration percentage (whether from automated or clinical results) is demonstrated. A strong correlation was found between the *AHI* and the total event duration percentage. Although the event durations are currently not considered important in the clinic, our results indicated that this parameter may be a new dimension for SAHS diagnosis or even a more efficient criterion.

## Limitations

While the proposed automated procedure for detecting sleep apnea-hypopnea syndrome (SAHS) events demonstrates strong statistical accuracy and efficiency, several limitations should be noted:

• **Event-by-event accuracy:** Although the overall apnea-hypopnea index (AHI) estimates are highly consistent with clinical reports, event-by-event agreement remains suboptimal. Occasional mismatches occur due to ambiguous event boundaries and overlapping physiological features.
• **Lack of patient-specific calibration:** The method employs fixed threshold values for all subjects. Variability in individual respiratory characteristics may affect detection accuracy. Incorporating adaptive or personalized thresholds could improve robustness.
• **Limited signal inputs:** This method uses only airflow and blood oxygen saturation signals. It excludes other physiological indicators such as EEG, ECG, or snoring, which are commonly employed in comprehensive SAHS assessment. Consequently, the method does not support subtype classification (e.g., central vs. obstructive apnea).
• **Device-specific validation:** The method was validated using data from a specific recording setup and software environment. Its generalizability to other hardware systems or signal formats remains to be verified.

These limitations highlight potential directions for future improvements, including multi-signal integration, individualized calibration strategies, and expanded cross-device validation.

## Ethics statements

This study was conducted in accordance with the Declaration of Helsinki and its amendments. The local Ethics Committee of the Eye & ENT Hospital of Fudan University confirmed that no additional ethical approval was required, as all procedures were part of routine clinical care. Informed consent was obtained from all participants prior to data collection.

## CRediT authorship contribution statement

**Yi Yu:** Methodology, Software, Data curation, Writing – original draft, Visualization, Funding acquisition. **Jing-Jing Huang:** Formal analysis, Validation, Writing – review & editing. **Hui Yang:** Supervision, Writing – review & editing. **Liu-Jie Ren:** Investigation, Supervision, Funding acquisition, Supervision.

## Declaration of competing interest

The authors declare that they have no known competing financial interests or personal relationships that could have appeared to influence the work reported in this paper.

## Data Availability

Data will be made available on request.

## Appendix Supplementary materials

Image, application 1
