# Supplementary material for: Automated diagnostic method for sleep apnea and hypopnea using overnight airflow and oxygen saturation
Source: MethodsX. 2025 Jul 27;15:103528. doi: 10.1016/j.mex.2025.103528 (PMC12355581; doi:10.1016/j.mex.2025.103528)
Supplement: Supplementary file 1 [file mmc1.docx]

# Appendix

A list of diagnosing results of 143 patients.

| ***Patient number*** | **Total**  **sleep**  **time, s** | **Apnea**  **Events**  **manual** | **Hypo-**  **Events**  **manual** | **Apnea**  **Events**  **automate** | **Hypo-**  **Events**  **automate** | **SpO2**  **Drop**  **Events** | **SAH**  **Events**  **manual** | **SAH**  **Events**  **automate** | **AHI**  **manual** | **AHI**  **automate** | **AHI**  **PSG**  **system** |
| --- | --- | --- | --- | --- | --- | --- | --- | --- | --- | --- | --- |
| *1* | 18930 | 6 | 0 | 6 | 7 | 9 | 6 | 13 | 1.14 | 2.47 | 0.4 |
| *2* | 26040 | 6 | 7 | 4 | 7 | 11 | 13 | 11 | 1.80 | 1.52 | 0.1 |
| *3* | 25980 | 14 | 1 | 14 | 0 | 1 | 15 | 14 | 2.08 | 1.94 | 1.1 |
| *4* | 34710 | 8 | 13 | 6 | 16 | 20 | 21 | 22 | 2.18 | 2.28 | 0.2 |
| *5* | 29460 | 18 | 2 | 6 | 5 | 9 | 20 | 11 | 2.44 | 1.34 | 0.4 |
| *6* | 29880 | 8 | 14 | 7 | 18 | 20 | 22 | 25 | 2.65 | 3.01 | 1.8 |
| *7* | 26640 | 11 | 12 | 9 | 4 | 8 | 23 | 13 | 3.11 | 1.76 | 0.7 |
| *8* | 33570 | 4 | 29 | 1 | 15 | 15 | 33 | 16 | 3.54 | 1.72 | 0.9 |
| *9* | 35370 | 24 | 16 | 18 | 20 | 32 | 40 | 38 | 4.07 | 3.87 | 1.8 |
| *10* | 37050 | 39 | 8 | 18 | 10 | 17 | 47 | 28 | 4.57 | 2.72 | 3.2 |
| *11* | 32610 | 41 | 10 | 29 | 18 | 39 | 51 | 47 | 5.63 | 5.19 | 1.9 |
| *12* | 18210 | 27 | 4 | 20 | 12 | 24 | 31 | 32 | 6.13 | 6.33 | 6 |
| *13* | 36120 | 60 | 11 | 45 | 33 | 56 | 71 | 78 | 7.08 | 7.77 | 7 |
| *14* | 33150 | 6 | 64 | 2 | 63 | 64 | 70 | 65 | 7.60 | 7.06 | 2.4 |
| *15* | 29760 | 27 | 39 | 12 | 97 | 159 | 66 | 109 | 7.98 | 13.19 | 7 |
| *16* | 23190 | 1 | 53 | 0 | 13 | 13 | 54 | 13 | 8.38 | 2.02 | 3.6 |
| *17* | 22230 | 41 | 17 | 40 | 5 | 43 | 58 | 45 | 9.39 | 7.29 | 4.8 |
| *18* | 28140 | 28 | 46 | 18 | 47 | 59 | 74 | 65 | 9.47 | 8.32 | 4.4 |
| *19* | 36060 | 45 | 53 | 26 | 47 | 65 | 98 | 73 | 9.78 | 7.29 | 3.1 |
| *20* | 27420 | 37 | 41 | 23 | 35 | 58 | 78 | 58 | 10.24 | 7.61 | 5.9 |
| *21* | 32730 | 96 | 15 | 67 | 47 | 100 | 111 | 114 | 12.21 | 12.54 | 6.3 |
| *22* | 29970 | 76 | 28 | 44 | 55 | 91 | 104 | 99 | 12.49 | 11.89 | 5.9 |
| *23* | 31470 | 103 | 9 | 106 | 8 | 56 | 112 | 114 | 12.81 | 13.04 | 15.5 |
| *24* | 17340 | 3 | 61 | 1 | 43 | 43 | 64 | 44 | 13.29 | 9.13 | 1.8 |
| *25* | 28440 | 47 | 62 | 79 | 46 | 88 | 109 | 125 | 13.80 | 15.82 | 6.5 |
| *26* | 33570 | 9 | 120 | 9 | 104 | 110 | 129 | 113 | 13.83 | 12.12 | 4.7 |
| *27* | 29670 | 65 | 59 | 62 | 62 | 122 | 124 | 124 | 15.05 | 15.05 | 9.2 |
| *28* | 25650 | 74 | 34 | 61 | 63 | 133 | 108 | 124 | 15.16 | 17.40 | 7.6 |
| *29* | 14340 | 32 | 37 | 8 | 56 | 61 | 69 | 64 | 17.32 | 16.07 | 5.5 |
| *30* | 38610 | 78 | 114 | 38 | 188 | 213 | 192 | 226 | 17.90 | 21.07 | 11.1 |
| *31* | 21510 | 29 | 82 | 19 | 95 | 112 | 111 | 114 | 18.58 | 19.08 | 9.4 |
| *32* | 35010 | 122 | 67 | 92 | 108 | 177 | 189 | 200 | 19.43 | 20.57 | 12.8 |
| *33* | 27210 | 72 | 75 | 63 | 83 | 189 | 147 | 146 | 19.45 | 19.32 | 15.8 |
| *34* | 31320 | 160 | 14 | 148 | 19 | 130 | 174 | 167 | 20.00 | 19.20 | 14.1 |
| *35* | 22290 | 50 | 74 | 62 | 58 | 267 | 124 | 120 | 20.03 | 19.38 | 12.8 |
| *36* | 26010 | 8 | 145 | 20 | 187 | 204 | 153 | 207 | 21.18 | 28.65 | 15 |
| *37* | 33630 | 36 | 162 | 33 | 144 | 175 | 198 | 177 | 21.20 | 18.95 | 8.7 |
| *38* | 30720 | 100 | 92 | 96 | 82 | 167 | 192 | 178 | 22.50 | 20.86 | 16.2 |
| *39* | 27330 | 142 | 34 | 109 | 49 | 145 | 176 | 158 | 23.18 | 20.81 | 15.6 |
| *40* | 24240 | 107 | 51 | 113 | 26 | 127 | 158 | 139 | 23.47 | 20.64 | 17.2 |
| *41* | 22500 | 11 | 137 | 1 | 155 | 155 | 148 | 156 | 23.68 | 24.96 | 6.5 |
| *42* | 32070 | 155 | 59 | 191 | 44 | 158 | 214 | 235 | 24.02 | 26.38 | 24.2 |
| *43* | 26310 | 110 | 76 | 83 | 97 | 171 | 186 | 180 | 25.45 | 24.63 | 13.7 |
| *44* | 31380 | 70 | 153 | 58 | 174 | 230 | 223 | 232 | 25.58 | 26.62 | 17 |
| *45* | 26580 | 73 | 121 | 96 | 89 | 180 | 194 | 185 | 26.28 | 25.06 | 21.9 |
| *46* | 35580 | 192 | 70 | 218 | 56 | 175 | 262 | 274 | 26.51 | 27.72 | 15.7 |
| *47* | 34770 | 221 | 36 | 244 | 25 | 169 | 257 | 269 | 26.61 | 27.85 | 12.9 |
| *48* | 35070 | 202 | 69 | 208 | 52 | 299 | 271 | 260 | 27.82 | 26.69 | 14.9 |
| *49* | 37560 | 216 | 85 | 166 | 101 | 227 | 301 | 267 | 28.85 | 25.59 | 16.4 |
| *50* | 21960 | 148 | 29 | 159 | 9 | 128 | 177 | 168 | 29.02 | 27.54 | 29.7 |
| *51* | 33780 | 235 | 42 | 216 | 53 | 218 | 277 | 269 | 29.52 | 28.67 | 24 |
| *52* | 28500 | 206 | 43 | 196 | 27 | 40 | 249 | 223 | 31.45 | 28.17 | 18.8 |
| *53* | 33900 | 207 | 92 | 198 | 77 | 244 | 299 | 275 | 31.75 | 29.20 | 20.3 |
| *54* | 29160 | 255 | 3 | 230 | 0 | 290 | 258 | 230 | 31.85 | 28.40 | 36.3 |
| *55* | 31080 | 235 | 42 | 228 | 50 | 219 | 277 | 278 | 32.08 | 32.20 | 27.8 |
| *56* | 17940 | 134 | 28 | 110 | 30 | 118 | 162 | 140 | 32.51 | 28.09 | 13.2 |
| *57* | 33570 | 221 | 86 | 212 | 83 | 282 | 307 | 295 | 32.92 | 31.64 | 20.9 |
| *58* | 29070 | 92 | 188 | 101 | 173 | 264 | 280 | 274 | 34.67 | 33.93 | 18.9 |
| *59* | 38550 | 240 | 132 | 224 | 125 | 322 | 372 | 349 | 34.74 | 32.59 | 22 |
| *60* | 35820 | 188 | 159 | 151 | 175 | 334 | 347 | 326 | 34.87 | 32.76 | 18.7 |
| *61* | 36000 | 134 | 238 | 150 | 190 | 324 | 372 | 340 | 37.20 | 34.00 | 16.9 |
| *62* | 34080 | 295 | 59 | 238 | 49 | 309 | 354 | 287 | 37.39 | 30.32 | 24.1 |
| *63* | 35460 | 263 | 115 | 281 | 90 | 342 | 378 | 371 | 38.38 | 37.66 | 31.6 |
| *64* | 36090 | 341 | 46 | 275 | 112 | 340 | 387 | 387 | 38.60 | 38.60 | 24.3 |
| *65* | 37590 | 396 | 12 | 359 | 36 | 272 | 408 | 395 | 39.07 | 37.83 | 41.3 |
| *66* | 32850 | 224 | 134 | 227 | 112 | 277 | 358 | 339 | 39.23 | 37.15 | 22 |
| *67* | 34650 | 211 | 172 | 192 | 203 | 365 | 383 | 395 | 39.79 | 41.04 | 29.3 |
| *68* | 25830 | 209 | 80 | 214 | 70 | 151 | 289 | 284 | 40.28 | 39.58 | 27.1 |
| *69* | 33360 | 370 | 4 | 346 | 8 | 522 | 374 | 354 | 40.36 | 38.20 | 62.5 |
| *70* | 26220 | 182 | 113 | 156 | 147 | 293 | 295 | 303 | 40.50 | 41.60 | 32.7 |
| *71* | 31110 | 267 | 86 | 252 | 128 | 372 | 353 | 380 | 40.85 | 43.97 | 26.7 |
| *72* | 33750 | 291 | 95 | 300 | 71 | 359 | 386 | 371 | 41.17 | 39.57 | 47.1 |
| *73* | 34620 | 388 | 24 | 316 | 59 | 256 | 412 | 375 | 42.84 | 38.99 | 27.2 |
| *74* | 21900 | 93 | 171 | 61 | 190 | 250 | 264 | 251 | 43.40 | 41.26 | 29.4 |
| *75* | 34470 | 85 | 335 | 58 | 351 | 400 | 420 | 409 | 43.86 | 42.72 | 27.3 |
| *76* | 29700 | 348 | 14 | 332 | 13 | 284 | 362 | 345 | 43.88 | 41.82 | 38.6 |
| *77* | 30810 | 278 | 112 | 235 | 146 | 356 | 390 | 381 | 45.57 | 44.52 | 31.4 |
| *78* | 30120 | 362 | 27 | 330 | 44 | 344 | 389 | 374 | 46.49 | 44.70 | 34.8 |
| *79* | 35040 | 336 | 119 | 269 | 110 | 299 | 455 | 379 | 46.75 | 38.94 | 41.5 |
| *80* | 27720 | 328 | 35 | 256 | 42 | 355 | 363 | 298 | 47.14 | 38.70 | 34.3 |
| *81* | 35490 | 467 | 2 | 433 | 1 | 460 | 469 | 434 | 47.57 | 44.02 | 39.1 |
| *82* | 36240 | 390 | 90 | 309 | 150 | 420 | 480 | 459 | 47.68 | 45.60 | 36.1 |
| *83* | 26160 | 330 | 17 | 324 | 10 | 290 | 347 | 334 | 47.75 | 45.96 | 40.7 |
| *84* | 30030 | 407 | 12 | 392 | 22 | 369 | 419 | 414 | 50.23 | 49.63 | 31.3 |
| *85* | 29190 | 404 | 6 | 391 | 0 | 353 | 410 | 391 | 50.57 | 48.22 | 56.4 |
| *86* | 37950 | 534 | 1 | 530 | 0 | 486 | 535 | 530 | 50.75 | 50.28 | 54.4 |
| *87* | 35460 | 498 | 3 | 507 | 2 | 504 | 501 | 509 | 50.86 | 51.68 | 47.5 |
| *88* | 15450 | 223 | 4 | 210 | 10 | 237 | 227 | 220 | 52.89 | 51.26 | 27.1 |
| *89* | 25590 | 375 | 5 | 385 | 7 | 331 | 380 | 392 | 53.46 | 55.15 | 45.5 |
| *90* | 37020 | 549 | 10 | 539 | 28 | 474 | 559 | 567 | 54.36 | 55.14 | 35.4 |
| *91* | 20220 | 236 | 72 | 233 | 66 | 284 | 308 | 299 | 54.84 | 53.23 | 23.9 |
| *92* | 19200 | 186 | 107 | 168 | 134 | 287 | 293 | 302 | 54.94 | 56.63 | 50.1 |
| *93* | 35280 | 484 | 58 | 474 | 73 | 520 | 542 | 547 | 55.31 | 55.82 | 54 |
| *94* | 40800 | 623 | 13 | 618 | 14 | 607 | 636 | 632 | 56.12 | 55.76 | 55.4 |
| *95* | 33600 | 525 | 1 | 523 | 4 | 532 | 526 | 527 | 56.36 | 56.46 | 59.1 |
| *96* | 31080 | 399 | 91 | 390 | 96 | 459 | 490 | 486 | 56.76 | 56.29 | 47.8 |
| *97* | 30960 | 432 | 57 | 412 | 74 | 443 | 489 | 486 | 56.86 | 56.51 | 26.9 |
| *98* | 26640 | 417 | 6 | 411 | 11 | 408 | 423 | 422 | 57.16 | 57.03 | 44.1 |
| *99* | 27780 | 353 | 90 | 348 | 95 | 385 | 443 | 443 | 57.41 | 57.41 | 49.8 |
| *100* | 34290 | 479 | 71 | 474 | 54 | 517 | 550 | 528 | 57.74 | 55.43 | 52.5 |
| *101* | 34140 | 520 | 34 | 532 | 4 | 542 | 554 | 536 | 58.42 | 56.52 | 39.9 |
| *102* | 32190 | 518 | 21 | 508 | 37 | 494 | 539 | 545 | 60.28 | 60.95 | 37.9 |
| *103* | 32280 | 294 | 247 | 165 | 347 | 488 | 541 | 512 | 60.33 | 57.10 | 52 |
| *104* | 34530 | 542 | 41 | 532 | 52 | 550 | 583 | 584 | 60.78 | 60.89 | 52.8 |
| *105* | 28740 | 427 | 68 | 373 | 104 | 424 | 495 | 477 | 62.00 | 59.75 | 36.6 |
| *106* | 29640 | 510 | 7 | 502 | 19 | 492 | 517 | 521 | 62.79 | 63.28 | 53.7 |
| *107* | 25050 | 271 | 167 | 198 | 244 | 427 | 438 | 442 | 62.95 | 63.52 | 32.3 |
| *108* | 24120 | 310 | 113 | 293 | 137 | 418 | 423 | 430 | 63.13 | 64.18 | 56 |
| *109* | 24750 | 425 | 12 | 387 | 24 | 391 | 437 | 411 | 63.56 | 59.78 | 57.2 |
| *110* | 33360 | 589 | 1 | 577 | 13 | 604 | 590 | 590 | 63.67 | 63.67 | 47.6 |
| *111* | 28650 | 530 | 5 | 504 | 29 | 478 | 535 | 533 | 67.23 | 66.97 | 57.5 |
| *112* | 32910 | 598 | 19 | 548 | 67 | 591 | 617 | 615 | 67.49 | 67.27 | 50.7 |
| *113* | 25830 | 489 | 3 | 478 | 0 | 0 | 492 | 478 | 68.57 | 66.62 | 48.5 |
| *114* | 25320 | 434 | 60 | 415 | 84 | 485 | 494 | 499 | 70.24 | 70.95 | 45.4 |
| *115* | 33720 | 601 | 61 | 559 | 41 | 192 | 662 | 600 | 70.68 | 64.06 | 59.6 |
| *116* | 34020 | 661 | 7 | 647 | 8 | 700 | 668 | 655 | 70.69 | 69.31 | 61 |
| *117* | 35130 | 682 | 9 | 646 | 27 | 689 | 691 | 673 | 70.81 | 68.97 | 57.5 |
| *118* | 35430 | 638 | 61 | 523 | 134 | 591 | 699 | 657 | 71.02 | 66.76 | 61.8 |
| *119* | 30840 | 596 | 16 | 581 | 34 | 571 | 612 | 615 | 71.44 | 71.79 | 67.2 |
| *120* | 28170 | 575 | 9 | 535 | 38 | 546 | 584 | 573 | 74.63 | 73.23 | 71.3 |
| *121* | 34560 | 705 | 15 | 661 | 55 | 665 | 720 | 716 | 75.00 | 74.58 | 52.3 |
| *122* | 28020 | 572 | 16 | 552 | 36 | 561 | 588 | 588 | 75.55 | 75.55 | 69.6 |
| *123* | 26340 | 516 | 58 | 471 | 84 | 477 | 574 | 555 | 78.45 | 75.85 | 73.4 |
| *124* | 36990 | 815 | 0 | 805 | 1 | 757 | 815 | 806 | 79.32 | 78.44 | 65.5 |
| *125* | 33360 | 694 | 54 | 652 | 33 | 722 | 748 | 685 | 80.72 | 73.92 | 61.5 |
| *126* | 34800 | 692 | 94 | 636 | 121 | 669 | 786 | 757 | 81.31 | 78.31 | 82.1 |
| *127* | 32970 | 743 | 5 | 684 | 22 | 722 | 748 | 706 | 81.67 | 77.09 | 57.9 |
| *128* | 24090 | 526 | 21 | 503 | 18 | 465 | 547 | 521 | 81.74 | 77.86 | 51.9 |
| *129* | 33510 | 754 | 12 | 750 | 14 | 720 | 766 | 764 | 82.29 | 82.08 | 75.7 |
| *130* | 35070 | 730 | 78 | 655 | 62 | 285 | 808 | 717 | 82.94 | 73.60 | 82.8 |
| *131* | 35880 | 713 | 114 | 700 | 109 | 788 | 827 | 809 | 82.98 | 81.17 | 78.5 |
| *132* | 33600 | 765 | 17 | 632 | 136 | 759 | 782 | 768 | 83.79 | 82.29 | 69.8 |
| *133* | 28860 | 671 | 13 | 608 | 29 | 624 | 684 | 637 | 85.32 | 79.46 | 72.3 |
| *134* | 34110 | 804 | 10 | 759 | 23 | 802 | 814 | 782 | 85.91 | 82.53 | 69.7 |
| *135* | 37350 | 901 | 4 | 873 | 32 | 889 | 905 | 905 | 87.23 | 87.23 | 80.6 |
| *136* | 34260 | 759 | 76 | 729 | 74 | 724 | 835 | 803 | 87.74 | 84.38 | 63.4 |
| *137* | 36360 | 900 | 13 | 868 | 24 | 760 | 913 | 892 | 90.40 | 88.32 | 73.6 |
| *138* | 36450 | 914 | 16 | 873 | 110 | 598 | 930 | 983 | 91.85 | 97.09 | 85.3 |
| *139* | 35400 | 678 | 230 | 543 | 378 | 865 | 908 | 921 | 92.34 | 93.66 | 84.3 |
| *140* | 33600 | 806 | 64 | 715 | 150 | 840 | 870 | 865 | 93.21 | 92.68 | 86.6 |
| *141* | 36030 | 907 | 35 | 758 | 160 | 943 | 942 | 918 | 94.12 | 91.72 | 89 |
| *142* | 38040 | 946 | 69 | 893 | 134 | 1014 | 1015 | 1027 | 96.06 | 97.19 | 98 |
| *143* | 29190 | 778 | 11 | 726 | 35 | 425 | 789 | 761 | 97.31 | 93.85 | 77.1 |
